# Elucidating anionic oxygen activity in lithium-rich layered oxides

**DOI:** 10.1038/s41467-018-03403-9

**Published:** 2018-03-05

**Authors:** Jing Xu, Meiling Sun, Ruimin Qiao, Sara E. Renfrew, Lu Ma, Tianpin Wu, Sooyeon Hwang, Dennis Nordlund, Dong Su, Khalil Amine, Jun Lu, Bryan D. McCloskey, Wanli Yang, Wei Tong

**Affiliations:** 1 Energy Storage and Distributed Resources Division, Lawrence Berkeley National Laboratory, Berkeley, CA 94720 USA; 2 Advanced Light Source, Lawrence Berkeley National Laboratory, Berkeley, CA 94720 USA; 3 Department of Chemical and Biomolecular Engineering, University of California, Berkeley, CA 94720 USA; 4 X-ray Sciences Division, Argonne National Laboratory, Argonne, IL 60439 USA; 5 Center for Functional Nanomaterials, Brookhaven National Laboratory, Upton, NY 11973 USA; 6 Stanford Synchrotron Radiation Lightsource, SLAC National Accelerator Laboratory, Menlo Park, CA 94025 USA; 7 Chemical Sciences and Engineering Division, Argonne National Laboratory, Argonne, IL 60439 USA

## Abstract

Recent research has explored combining conventional transition-metal redox with anionic lattice oxygen redox as a new and exciting direction to search for high-capacity lithium-ion cathodes. Here, we probe the poorly understood electrochemical activity of anionic oxygen from a material perspective by elucidating the effect of the transition metal on oxygen redox activity. We study two lithium-rich layered oxides, specifically lithium nickel metal oxides where metal is either manganese or ruthenium, which possess a similar structure and discharge characteristics, but exhibit distinctly different charge profiles. By combining X-ray spectroscopy with operando differential electrochemical mass spectrometry, we reveal completely different oxygen redox activity in each material, likely resulting from the different interaction between the lattice oxygen and transition metals. This work provides additional insights into the complex mechanism of oxygen redox and development of advanced high-capacity lithium-ion cathodes.

## Abstract (web summary)

A reversible oxygen redox process contributes extra capacity and understanding this behavior is of high importance. Here, aided by resonant inelastic X-ray scattering, the authors reveal the distinctive anionic oxygen activity of battery electrodes with different transition metals.

## Introduction

Rechargeable Li-ion battery technology has become the dominant power source for portable electronics, and is now on the verge of enabling widespread electric vehicle (EV) adoption.^1–4^ Nowadays, tremendous research efforts are being devoted to pushing the practical capacity of layered lithium transition-metal oxides, LiTMO_2_ (TM = Ni, Mn, Co, Al), close to their theoretical values.^5–7^ Meanwhile, a reversible capacity approaching 300 mAh g^–1^ was demonstrated by a modified version of a traditional layered oxide^8^, often reported by a formula of xLi_2_MnO_3_ · (1–x)Li(Mn, Ni, Co)O_2_^9–11^ or Li_1+x_(Mn, Ni, Co)_1–*x*_O_2_^12–15^.

This family of Li-rich layered oxides always demonstrates an exceptionally higher capacity (typically >250 mAh g^–1^) than traditional stoichiometric layered oxides (e.g., <190 mAh g^–1^ for LiCoO_2_).^16–22^ Such a high capacity is far beyond the theoretical capacity originating solely from Ni and Co redox (i.e., 127 mAh g^–1^ for Ni redox in Li_1.2_Ni_0.2_Mn_0.6_O_2_). Lattice oxygen is typically believed to compensate additional charge beyond TM redox upon the simultaneous removal of Li^+^. However, various mechanisms have been proposed regarding the precise nature of lattice oxygen participation in the active material electrochemistry. For example, O_2_ and/or CO_2_ gas evolution is known to occur in combination with oxygen vacancies at the particle surface.^16, 23–25^ Meanwhile, Mn^4+^ reduction to Mn^3+^ induced by electrode-electrolyte reactions and/or oxygen removal,^18, 26–28^ electrochemical reduction of O_2_ molecules generated on the initial charge^29^, and reaction of the organic electrolyte with Li_2_O formed during the extended high-voltage plateau^30^ were proposed to occur during subsequent electrochemical cycling.^31^ Recently, Koga et al. described two separate mechanisms for lattice oxygen participation during electrochemical cycling: irreversible oxygen loss at the surface and reversible redox of lattice oxygen within the bulk.^31, 32^

These studies raised significant interest in further verifying the reversible anionic oxygen redox because materials in which it is active could provide substantially higher capacity than conventional layered oxides.^33–38^ Extra capacity has been demonstrated in a series of Li_2_TMO_3_ compounds, particularly with *4d* and *5d* transition metals, in which the strong TM-O hybridization was believed to promote the reversible oxygen redox, i.e., O^2–^ → O_2_^n–^ (*n* = 1, 2, or 3).^39–44^ More specifically, formation of localized electron holes on oxygen that is coordinated by Mn and Li was proposed to occur upon Li removal in Li-, Mn-rich layered oxides.^45, 46^ Computational studies revealed the specific Li-O-Li configuration in Li-excess metal oxides holds the key to activate anionic oxygen redox by enabling the labile oxygen electrons.^47^ The above mentioned models consider different transition metals (i.e., *3d*, *4d*, and *5d*) that have different preference for TM-O hybridization might make the complex anionic oxygen redox even more elusive. Fundamental understanding of anionic oxygen redox is of critical importance to propose effective material design strategy to develop novel materials that harness active oxygen redox.

In this work, we aim to probe the electrochemical activity of anionic oxygen in Li-rich layered oxides by focusing on the effect of transition-metal species, Mn and Ru, representing *3d* and *4d* TM, on the oxygen redox process. Both Li-rich layered oxides, Li_1.2_Ni_0.2_TM_0.6_O_2_ (TM = Mn, Ru), possess a similar crystal structure and enable a similar amount of Li removal and uptake during the charge–discharge process, but with significantly different charge profiles characterized by the existence of a voltage plateau at 4.55 V in the Mn-based material, but not the Ru-based one. Here, we perform a comprehensive study to capture the oxygen activity, which could range from O^2–^ in the crystal lattice to O^0^ in the extreme case of O_2_ gassing, by combining a suite of soft X-ray spectroscopy techniques with in situ differential electrochemical spectrometry (DEMS). We observe completely different oxygen behavior in the two materials, the sharp contrast between them providing strong experimental evidence for a particularly large reversible anionic oxygen redox observed in the Mn-based material, but not the Ru-based one. We believe these findings will provide additional insights into the complex oxygen redox mechanism and development of advanced high-capacity Li-ion cathodes. Moreover, this work demonstrates an explicit example of the employment of resonant inelastic X-ray scattering (RIXS) technique in understanding solid-state oxygen redox processes for energy storage and conversion applications.

## Results

### Structural characterization of pristine Li_1.2_Ni_0.2_Mn_0.6_O_2_ and Li_1.2_Ni_0.2_Ru_0.6_O_2_

**Fig. 1:**
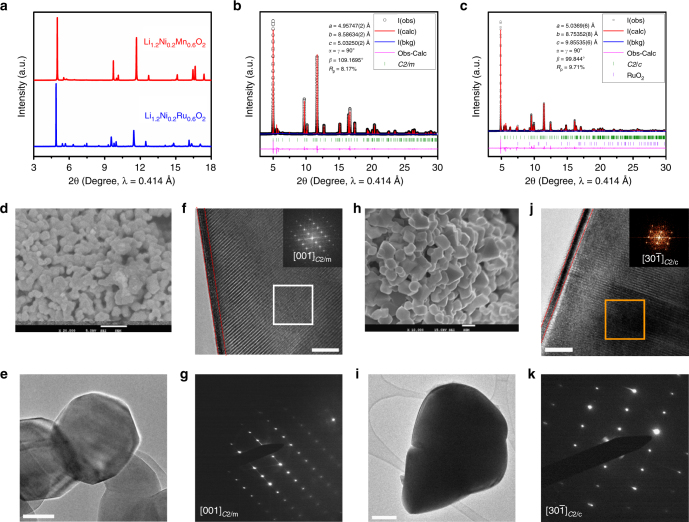
Structural characterization of pristine Li_1.2_Ni_0.2_Mn_0.6_O_2_ (LNMO) and Li_1.2_Ni_0.2_Ru_0.6_O_2_ (LNRO). **a** Synchrotron XRD patterns, showing a similar crystal structure between these two compounds; XRD Rietveld refinement of **b** LNMO based on monoclinic *C*2/*m* and **c** LNRO based on monoclinic *C*2/*c*; **d** scanning electron microscopy (SEM) image of LNMO, the scale bar is 1 μm; **e**, **f** high-resolution transmission electron microscopy (HRTEM) images of LNMO with fast Fourier transform (FTT) of the selected area, the scale bar in (e) and (f) is 50 and 5 nm, respectively; **g** electron diffraction (ED) pattern for LNMO; **h** SEM image of LNRO, the scale bar is 1 μm; **i**, **j** HRTEM images of LNRO with FTT of the selected area, the scale bar in (**i**) and (**j**) is 100 and 2 nm, respectively; **k** ED pattern for LNRO

The two compounds, Li_1.2_Ni_0.2_Mn_0.6_O_2_ and Li_1.2_Ni_0.2_Ru_0.6_O_2_, henceforth denoted as LNMO and LNRO, respectively, were synthesized by conventional solid-state reaction method. Both LNMO and LNRO exhibit similar X-ray diffraction (XRD) patterns (Fig. 1a). Most of the characteristic XRD peaks can be indexed based on *R*$\bar 3$*m* symmetry (α-NaFeO_2_), except those in the 2θ region of 5 to 8°, corresponding to 19 and 31° (CuK_*α*_ radiation, 1.5406 Å). These extra peaks are typically assigned to the superstructure peaks originating from Li and TM cation ordering on the TM layer in monoclinic Li_2_TMO_3_ (TM = Mn, Ru), being more clearly observed if its chemical formula is rewritten as Li[Li_1/3_TM_2/3_]O_2_. As such, the monoclinic phase, Li_2_MnO_3_, belongs to *C*2/*m* space group, while, Li_2_RuO_3_ was reported to be either *C*2/*c* or *P*21/*m*.^48, 49^ Such structural distortion could be resulted by a small lattice energy difference. To further confirm the similar crystal structure between LNMO and LNRO, high-resolution synchrotron XRD patterns were refined by the conventional Rietveld method (Fig. 1b, c). Our refinements were performed based on *R*$\bar 3$*m* and *C*2/*m*, *C*2/*c* for LNMO, LNRO, respectively. A good fit was obtained for LNMO for both *C*2/*m* (Fig. 1b) and *R*$\bar 3$*m* (Supplementary Fig. 1a). For LNRO refinement, *C*2/*c* symmetry gave a better fit (Fig. 1c) than *R*$\bar 3$*m* symmetry (Supplementary Fig. 1b). The refined structural parameters of LNMO and LNRO are given in Supplementary Table 1, 2, respectively. Rietveld refinement results suggest the as-produced LNMO and LNRO samples fit the structural model of monoclinic solid solution, though the nanocomposite concept concerning the mixture of layered LiNiO_2_ (*R*$\bar 3$*m*) and monoclinic Li_2_RuO_3_ (*C*2/*c*) cannot be excluded.

Scanning electron microscopy (SEM) and high-resolution transmission electron microscopy (HRTEM) were used to further verify the morphology and crystal structure. Overall, both exhibited a similar morphology, composed of agglomerated primary particles, as observed in SEM images (Fig. 1d, h). A number of particles were examined using HRTEM and representative images are shown in Fig. 1e–g, i–k. The LNMO sample has a slightly smaller primary particle size than the LNRO sample, both being a few hundred nanometers. They both exhibit a well-defined layered structure with a thin layer (~1.5 nm) of spinel and/or rocksalt phase on the surface. Both electron diffraction (ED) patterns and fast Fourier transformation results along the [001] and [30$\bar 1$] zone axis of *C*2/*m* and *C*2/*c* from LNMO and LNRO particle showed high structural consistency. In contrast, an ED pattern from a LNRO particle is inconsistent with that simulated based on *P*21/*m* symmetry, confirming that our LNRO sample belongs to *C*2/*c* space group. Therefore, a similar crystal structure and morphology between the two samples was confirmed using combined synchrotron XRD, TEM, and SEM techniques.

### First charge–discharge characteristics of LNMO and LNRO

**Fig. 2:**
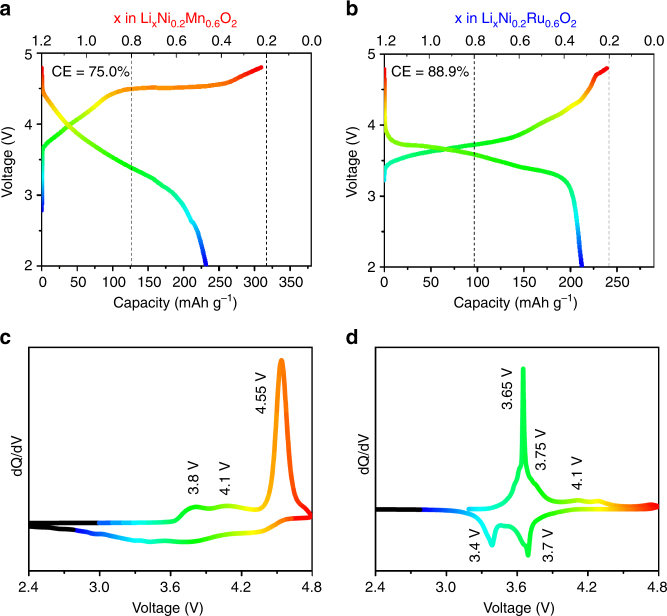
First charge–discharge characteristics of LNMO and LNRO. The first cycle voltage profile of **a** LNMO and **b** LNRO; differential capacity (d*Q*/d*V*) plot of **c** LNMO and **d** LNRO. Cells were cycled between 4.8 and 2.0 V at a current density of 5 mA g^–1^ at room temperature

The first cycle voltage profiles are shown in Fig. 2a, b. The as-produced LNRO sample demonstrated an electrochemical activity, characterized by a charge capacity of 239 mAh g^–1^ and a discharge capacity of 213 mAh g^−1^, leading to a coulombic efficiency of 88.9%. In comparison, LNMO sample exhibited a charge capacity of 310 mAh g^–1^ and a discharge capacity of 232 mAh g^–1^ with a coulombic efficiency of 75.0%. With respect to the theoretical capacity of 238 mAh g^–1^ and 318 mAh g^–1^ based on 1 Li^+^ for LNRO and LNMO, this corresponds to 1.01 Li^+^ removal/0.89 Li^+^ uptake for LNRO and 0.97/0.73 Li^+^ for LNMO, respectively. From this perspective, a common feature of about 1 Li^+^ removal during the charge was revealed for both compounds. Of note, the last 0.2 Li was not removed from both compounds in this study, additional studies regarding the reversible extraction of the final 0.2 Li are needed to understand the origin of this limitation (outside the scope of this study).

Despite this similarity, these two compounds exhibited noticeably different charge characteristics. Similar to other reported Li, Mn-rich layered oxides, LNMO exhibited a long plateau at 4.55 V in the high-voltage region, often referred to as the electrochemical activation process. In contrast, no obvious voltage plateau was observed for LNRO, instead, the charge profile of LNRO was a sloping curve with most of the capacity extracted below 4.5 V. This difference was even more pronounced in the differential capacity (d*Q*/d*V*) plots (Fig. 2c). The charge profile of LNMO was characterized by a strong anodic peak at 4.55 V, corresponding to the extended voltage plateau, as well as two weak anodic peaks around 3.8 and 4.1 V. In comparison, the strong anodic peak in the high-voltage region was absent in the charge profile of LNRO (Fig. 2d), and instead, a relatively strong peak at 3.65 V was observed along with two minor peaks at 3.75 and 4.1 V. The corresponding cathodic peaks, which were clearly visible in the LNRO, unlike the LNMO, were located around 3.7 and 3.4 V. By comparing these features to the Li_2_RuO_3_ base compounds^39, 40, 44, 50, 51^, it is likely that the oxidation/reduction couple around 3.65/3.4 V is related to Ru redox, but the shape of the curves suggests that Ni2+ and Ru4+ oxidation might be superimposed.

The structural and electrochemical characterization of LNMO and LNRO compounds reveal three interesting observations: (1) the compounds possess a similar crystal structure, (2) they allow a similar amount of Li removal on the initial charge, but (3) they have remarkably different charge profiles (voltage plateau at 4.55 V for LNMO vs. a sloping profile in LNRO). Given these unique features, LNMO and LNRO might be suitable model compounds to investigate the underlying oxygen activation mechanisms in the high-voltage region.

### Gas evolution of LNMO and LNRO by operando DEMS

**Fig. 3:**
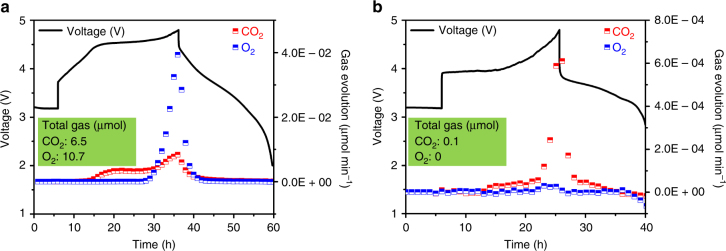
Gas evolution of LNMO and LNRO by operando DEMS. The first cycle voltage profiles and gas evolution rates of **a** LNMO and **b** LNRO. The total active cathode material used for the measurement was 32.9 mg LNMO (387 μmol) and 28.6 mg LNRO (253 μmol). Cells were cycled between 4.8 and 2.0 V, at a current of 10 mA g^–1^

We first used operando DEMS to monitor any oxygen evolution stemming from the 2 O^2–^ → O_2_^0^ + 4 e^–^ compensation reaction and quantify the irreversible loss of lattice oxygen as gaseous products, thus probe the extent of irreversibility of any oxygen participation in the charging process. The operando DEMS results (Fig. 3) were collected at a current density of 10 mA g^–1^ with an electrode loading of ~30 mg cm^–2^. The higher loading and larger current density accounted for a larger overpotential observed at the beginning of charge for LNRO and LNMO. The polarization was particularly pronounced for LNRO because of its relatively large particle size. Trace amounts (<0.1 nmol min^–1^) of H_2_, C_2_H_4_, CH_4_, and CO were detected at the beginning of charging, but are not shown here for clarity. The LNMO electrode tested in the operando DEMS cell demonstrated a charge capacity of 302 mAh g^–1^ and a discharge capacity of 234 mAh g^–1^, consistent with those obtained in our regular electrochemical cells. For LNMO (Fig. 3a), CO_2_ began to evolve at 4.0 V, the evolution rate moderately increasing with potential and reaching a maximum at 4.8 V. Exhibiting drastically different behavior, O_2_ evolution was not detected until 4.5 V, the O_2_ evolution rate dramatically increasing with potential, reaching a rate of ~4 10^–2^ μmol min^–1^ at 4.8 V. The cumulative CO_2_ and O_2_ detected from LNMO during the first cycle was 6.5 and 10.7 μmol, respectively.

If we assume O_2_ evolution originates solely from the active electrode (rather than electrolyte decomposition), 10.7 μmol total O_2_ evolution corresponds to 2.8% of the lattice oxygen in LNMO (32.9 mg, equivalent to 387 μmol). Taking 4 e^–^/O_2_, as would be expected for O_2_ release from lattice oxygen, the total irreversible capacity associated with this oxygen loss is 35 mAh g^–1^, which can account for slightly less than half of the total irreversible capacity (78 mAh g^–1^) on the first charge. Electrolyte (EC/DEC) is electrochemically stable until ~4.8 V with a blank electrode composed of conductive carbon and PVDF.^52, 53^ We do not attempt to ascribe a capacity to the CO_2_ evolution, as CO_2_ is likely evolved from surface Li_2_CO_3_ that remains after synthesis. Of note, any non-volatile electrolyte decomposition products that cannot be detected using DEMS could contribute to the irreversible capacity on charge. The gas evolved during the second cycle was composed of 0.9 μmol CO_2_ with no detectable O_2_.

In stark contrast to the substantial gas evolution from LNMO, LNRO evolved a minimal amount of CO_2_ and almost no O_2_ (Fig. 3b). Note that the LNRO gas evolution is plotted on a different scale than that of LNMO for clarity. For LNRO, no appreciable O_2_ evolution was detected in the charging process, with the evolution rate remaining below 10^–2^ nmol min^–1^, even at 4.8 V. Similar to LNMO, CO_2_ gas evolution began at 4.0 V and increased with charge potential, however, the rate of CO_2_ evolution was considerably less in LNRO, with only 0.1 μmol total CO_2_ evolved in the entire first cycle. This implies less residual surface carbonate and/or a different reactivity of LNRO with the carbonate electrolyte, likely due to its slightly large particle size compared to LNMO. In contrast to LNMO, with no O_2_ detected from LNRO, we do not ascribe any irreversible charge capacity to oxygen participation via oxygen loss.

The operando DEMS measurements revealed completely different outgassing behaviors in LNMO and LNRO, when they were cycled under identical conditions. Even when LNRO was charged to a higher potential (5.0 V), no O_2_, but a moderate increase in CO_2_ evolution (0.6 μmol) was detected, which was significantly lower compared to the total gas evolution from LNMO (Supplementary Fig. 2). Such drastic differences indicate these two compounds might exhibit significantly different oxygen activity in the solid-state as well. Therefore, we probed the electronic structures of transition metals and oxygen using elemental sensitive X-ray spectroscopy techniques to study the electrochemical redox change during the electrochemical reaction.

### Electronic structures of Ni and O as probed by sXAS

**Fig. 4:**
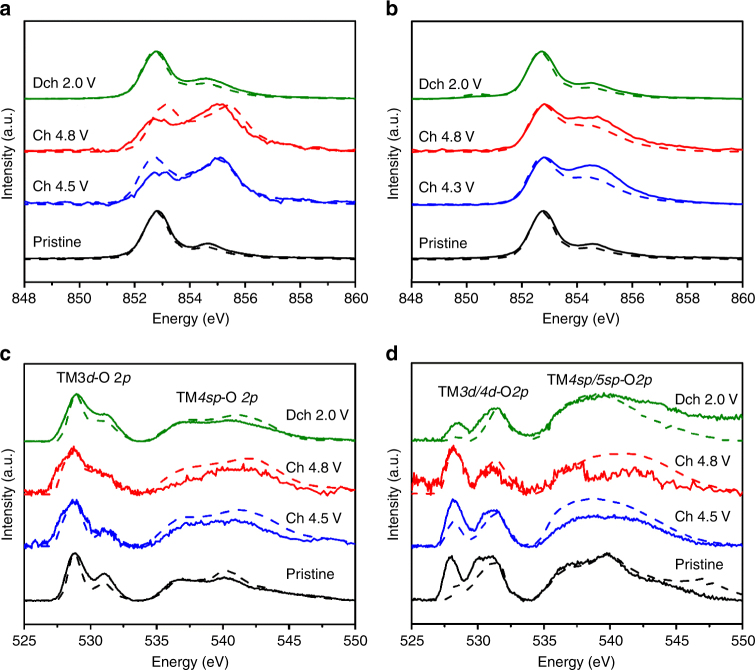
Electronic structures of Ni and O as probed by sXAS. sXAS Ni L3-edge spectra of **a** LNMO and **b** LNRO electrodes; sXAS O K-edge spectra of **c** LNMO and **d** LNRO electrodes in FY and TEY modes at different states of charge. Solid and dash line indicate FY and TEY mode, respectively

Soft X-ray absorption spectroscopy (sXAS) naturally provides elemental and orbital sensitivity to the redox states in battery electrodes with varied depth sensitivities.^54^ Due to the different mean free path of electrons and photons, total electron yield (TEY) and fluorescence yield (FY) modes yield chemical information at a depth of 10 nm and ∼100 nm from the particle surface, respectively. As shown in Fig. 4a, b, Ni L3-edge at 852–856 eV exhibits splitting features at high and low energy. The strong Ni L3-edge feature at 852.9 eV is from Ni^2+^, 855.1 eV from Ni^4+^, while Ni^3+^ is located at a slightly lower energy range with multiple features.^54, 55^ It is clearly shown that Ni is in its 2+ oxidation state in both pristine LNMO and LNRO. Upon charging, the general increase in Ni L3-edge peak in the high-energy range suggests Ni oxidation. The strong 855.1 eV peak of the charged LNMO at 4.5 V suggests Ni^4+^ state, while the relatively broad shoulder feature of the charged LNRO indicates a mixed valence of Ni^3+^ and Ni^4+^. Further increasing charging voltage to 4.8 V did not lead to an obvious change in Ni oxidation state, suggesting the completion of Ni redox by 4.5 V and 4.3 V for LNMO and LNRO, respectively. The Ni valence state reverts back to its divalent state after the first discharge, as the discharged material spectra are similar to those of the pristine material. Comparison between FY and TEY mode for both compounds revealed a slight Ni reduction at the surface of the charged electrodes, as evidenced by a slight decrease in the relative intensity of the TEY Ni L3-edge feature compared to the analogous FY spectra at high energy. No major change in Mn L3-edge (Supplementary Fig. 3a) was detected, but a small reduction after discharge was revealed.

Variation in transition-metal redox during the electrochemical reaction is inextricably linked to the hybridization between TM*3d/4d* and O*2p* orbitals. In an attempt to examine the electronic states of O at various charging states, we collected O K-edge sXAS spectra (Fig. 4c, d), which are characterized by the pre-edge peaks between 525 and 535 eV and a broad peak above 535 eV, corresponding to the hybridization of TM*3d/4d*-O*2p* and TM*4sp/5sp*-O*2p*, respectively. The pre-edge features can be further distinguished by two peaks, characterizing the excitation from the O*2p* orbital to unoccupied *t*_*2g*_ (<530 eV) and *e*_*g*_ orbitals (>530 eV) of the TM, accordingly. The new low-energy shoulder at 527.8 eV in the spectra of 4.5, 4.8 V charged LNMO is from the Ni^4+^ state.^55, 56^ Estimates of the average electron hole distribution and effective charge on oxygen anions have been gleaned by measuring the absorption intensity of sXAS.^26, 45, 46, 57, 58^ However, it is difficult to differentiate TM-O hybridization from the intrinsic oxygen state because the hybridization peaks are overlapping with the O*2p* states (Supplementary Fig. 3b). Furthermore, the O-K pre-edge intensity change upon electrochemical cycling is expected, because Li extraction often leads to more covalent nature, thus enhances the TM-O hybridization feature, regardless of oxygen redox. Indeed, such lineshape and intensity changes were observed in other battery electrodes, e.g., spinel^55^ and olivine^59^ materials. Therefore, we decided not to discuss the oxygen redox based on O-K sXAS. The pre-edge peak above 530 eV in sXAS O K-edge of LNRO (Fig. 4d) at the pristine and discharged states was more pronounced compared to that of LNMO, due to the different hybridization features between Ru-O^60^ and Mn-O^28^. Additionally, the difference between the pristine and discharged LNRO samples is likely due to the formation of surface species, which is often different for materials with different surface reactivity.

### Electronic structures of O as probed by RIXS

Effective probe of the intrinsic lattice oxygen activity calls for new techniques that could provide more sensitivity to the chemical states of lattice oxygen. Therefore, we employed RIXS technique to further reveal the decay information at each sXAS excitation energy. RIXS can be considered as a “further resolved” sXAS decay process by providing detailed information on energy distribution of the fluorescence signals.^61, 62^ The new information on the energy of the outgoing (emission) photons further resolves the electron states that are involved in the different low-energy excitations, thus providing much more sensitivity and clarification to define the chemical state of oxygen.

**Fig. 5:**
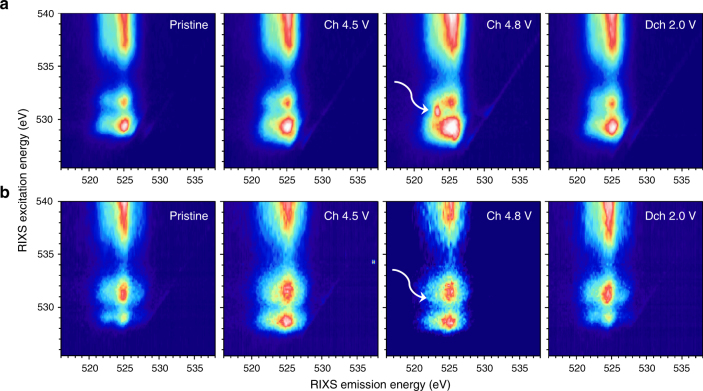
Electronic structures of O as probed by RIXS. O K-edge RIXS maps of **a** LNMO and **b** LNRO electrodes at different states of charge. The while arrow points to the specific oxygen redox state that is absent in LNRO

The RIXS intensity presented in Fig. 5 is plotted against the excitation energy (*y*-axis, same as *x*-axis in sXAS) and emission energy (*x*-axis). In the O K-edge maps, RIXS decay channels are dominated by fluorescence features (vertical stripes in the maps) that have fixed emission energy around 525 eV. These vertical RIXS stripes along the 525 eV emission energy have been extensively studied in transition-metal oxides and are characteristic of the TM-O hybridization and general O^2−^-2p bands.^63^ Consistent with sXAS spectra, the RIXS map of O K-edge is composed of a dispersed stripe feature, corresponding to TM*4sp/5sp*-O*2p* hybridization at excitation energies above 535 eV, and two small features at excitation energies below 535 eV, which are associated with TM*3d/4d*-O*2p* hybridization. For LNMO electrodes (Fig. 5a), one relatively large fluorescence feature was observed at an excitation energy of ~529.2 eV (*t*_*2g*_ orbitals) along with a relatively small feature at ~531.5 eV (*e*_*g*_ orbitals) in the pristine state. Upon charge, the 529.2 eV feature tended to grow due to its more covalent nature (corresponding to Ni oxidation), which was consistent with the enhancement of the sXAS peak below 530 eV during charge (Fig. 4c).

When the LNMO was charged to 4.8 V, a striking RIXS feature appears (marked by white arrow in Fig. 5a) at excitation and emission energies of 530.8 and 523.75 eV, respectively. The critical information that is clearly revealed here is the emission energy of this additional oxygen feature was lower than that of the standard fluorescence features originating from the TM-O hybridization as discussed above. More importantly, this sharp RIXS feature disappeared after 2.0 V discharge, indicating this feature is a unique fingerprint of the oxygen state change upon electrochemical cycling. Although the ultimate understanding of this RIXS feature requires sophisticated theoretical RIXS interpretation, which is an ongoing effort and out of the scope of this experimental report, we note that such RIXS feature has been observed in non-O^2–^ species, e.g., O_2_ gas^64, 65^. The standard features in the RIXS maps of LNRO O-K edge are also along 525 eV emission energy, similar to those of LNMO. The different feature profile with excitation energies of 528.5 and 531.1 eV is due to the TM-O hybridization, consistent with the lineshape difference in sXAS (Fig. 4c, d). However, in sharp contrast to that of LNMO, at 4.8 V charge, no striking feature was observed in LNRO (Fig. 5b). This unique striking feature that appears at 530.8 eV excitation and 523.75 eV emission energies is a direct evidence of the oxygen state change (oxygen redox) in the electrochemistry of LNMO. Therefore, no oxygen participates in the electrochemistry of LNRO, and hence Ru must be electrochemically active (as confirmed later).

### Electronic structure of Ru as probed by in situ XAS

**Fig. 6:**
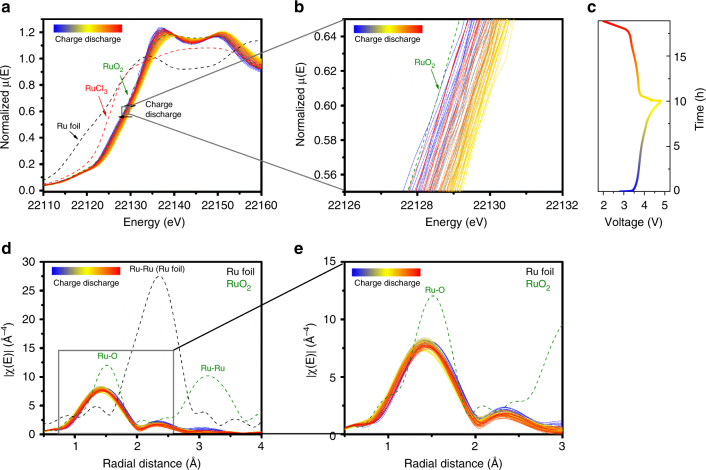
Electronic structure of Ru as probed by in situ XAS. In situ **a**, **b** XANES, **c** voltage profile, **d**, **e** EXAFS of Ru K-edge of LNRO during the first cycle. The in situ cell was charged at C/10 and discharged at C/7

Due to the high excitation energy of Ru, RIXS maps of Ru L-edge were not collected. As discussed above, reversible Ru redox is anticipated to participate in the electrochemistry of LNRO because the RIXS maps of O K-edge imply that lattice oxygen is not contributing to charge compensation. To verify our hypothesis, in situ hard XAS spectra (Fig. 6) were collected on the Ru K-edge during the first cycle of LNRO. Compared to the X-ray absorption near-edge spectra (XANES) of the reference compounds, including RuO_2_ (4+), RuCl_3_ (3+), and Ru metal (0), the oxidation state of Ru was determined to be 4+ in pristine LNRO (Fig. 6a). Upon charging, the Ru edge gradually shifted from its pristine state to higher energies, indicating Ru was oxidized. The oxidation state of Ru at the end of charge was estimated to be 5+ by measuring the energy shift at half the maximal amplitude of XANES. The energy of Ru K-edge XANES for pristine and charged state was 22126.8 and 22128.5 eV, showing an increase of 1.7 eV in energy. Such energy shift is in accordance with that (1.5 eV) for SuRuO_3_ and Sr_2_GdRuO_6_ as the oxidation state of octahedrally coordinated Ru increases from 4+ to 5+.^66^ Meanwhile, the Ru edge moved back to the lower energy and almost to its original valence state upon discharge.

On the other hand, extended X-ray absorption fine structure (EXAFS) shown in Fig. 6b exhibits two distinct shells around 1.4 and 2.3 Å, corresponding to Ru–O and Ru–TM interactions, respectively. The two shells shifted slightly to smaller distances as a result of Ru oxidation during the charge process, then reverted back to their pristine distances after discharge, indicating the reversible nature of the Ru redox process during LNRO cycling. In further support of the Ru redox reversibility, no obvious change in amplitude of the Ru-O and Ru-TM shells was observed, revealing no change in Ru coordination during the electrochemical process. In situ XAS results clearly demonstrated the electrochemical activity of Ru and its good reversibility during electrochemical cycling, lending support to our earlier hypothesis that lattice oxygen redox need not participate in charge compensation during Li insertion/extraction.

## Discussion

We clearly showed that the replacement of *3d* Mn by *4d* Ru substantially influenced the charge profile, and using a variety of spectroscopic techniques, we confirmed that this influence is primarily related to the different redox processes occurring in each material. Our discussion mainly focuses on the potential impact of the transition metal on oxygen redox activity. As opposed to Mn in LNMO, Ru is electrochemically active in LNRO. Despite the difficulty in determining the final valence state of Ru, Ru was indirectly verified to be at pentavalent state after charge. Given a reversible capacity of 213 mAh g^–1^ for the LNRO sample (corresponding to 0.9 Li^+^ based on the theoretical redox of Ni and Ru), it is reasonable to conclude that anionic oxygen redox is not needed for charge compensation, and hence is not active in the LNRO sample. In contrast, the reversible capacity of 232 mAh g^–1^ for the LNMO sample, far beyond its theoretical value (127 mAh g^–1^) solely relying on Ni redox, implies the necessity of lattice oxygen redox in the electrochemistry. Therefore, the distinct feature observed in the O K-edge RIXS map of LNMO (but not LNRO) at 4.8 V charge, coupled with the fact that capacity was observed beyond that expected from the full transition-metal redox, is direct experimental evidence of reversible anionic oxygen redox in LNMO. The selection of LNMO and LNRO for anionic oxygen activity studies is largely due to the similar crystal structure as well as # of Li^+^/e^−^ transfer during the charge–discharge process. However, from the perspective of their redox activity, Mn4+ is difficult to be further oxidized, whereas Ru4+ is easily oxidized to Ru5+. Therefore, comparison of other Li-rich metal oxides that integrate Ni and a second TM with no further oxidation beyond 4+ (e.g., Zr4+ or Pd4+) will be valuable as they can form monoclinic Li_2_ZrO_3_ (*C*2/*c*)^67^ and Li_2_PdO_3_ (*C*2/*m*)^68^, to the best of our knowledge, the electrochemistry of which are not well studied yet.

In contrast to the previously reported anionic oxygen redox in Li-, Mn-rich layered oxide, oxygen redox appeared to be suppressed in the Li-rich LNRO. Of note, no additional redox activity/capacity was revealed at a higher charge cutoff voltage (5.0 V) (Supplementary Fig. 4). Similar absence of anionic oxygen activity was previously observed for Li_4_NiTeO_6_.^69^ Participation of electrons from anionic oxygen can be traced to the electronic structure of metal and oxygen (M-O) bonding, including the unique Li-O-Li configuration in Li-rich metal oxides, because certain metal bonding (e.g., Li-O-Li) can lead to unhybridized O*2p* states that promotes the participation of electrons from oxygen in charge compensation.^47^ We note the Li content in our LNRO sample is not sufficient to allow all O to exist in a Li-O-Li configuration, which might subsequently influence the activation of anionic oxygen in LNRO. Additionally, oxygen activation might also be inhibited by kinetic factors (e.g., particle size, cycling temperature etc.).^70^ However, the active anionic oxygen in LNMO, with the same Li-O-Li configuration as LNRO, confirms the crucial role that the transition metal plays in the activation of anionic oxygen. A competition between the cationic and anionic redox or inhibition of one by the other seems possible. Based on previous studies on Ni and Ru redox in structurally relevant Li-rich metal oxides, the Ru^4+^/Ru^5+^ redox seems occur at a lower voltage compared to that of Ni^2+^/Ni^3+^/Ni^4+^. In Li_2_Ru_1–*x*_TM_*x*_O_3_ (TM = Mn, Ti, and Sn), these transition metals are electrochemically inactive and difficult to oxidize further up to 5+. Therefore, there are no sufficient electrons from TM beyond Ru to balance the amount of Li extraction, driving the O participation in the electrochemistry. In LNRO, both Ni and Ru are electrochemically active, full oxidation of both accounts for Li removal. The absence of anionic oxygen activity during the removal of 1 Li from LNRO suggests it is more difficult for the participation of electrons from oxygen than TM, because O redox varies with TM combinations, e.g., 4.5 V after Ni redox in LNMO vs. 4.3 V after Ru redox in Li_2_Ru_1–*x*_TM_*x*_O_3_. More transition-metal combinations, with a rational design by taking into account the transition-metal redox, should be explored in these family of Li-rich layered oxides, as well as other Li-rich metal oxides that crystallize in different crystal systems, such as disordered rock salts, to further reveal the impact of O local environment in anionic oxygen activity. Such fundamental studies should also be extended to Li-rich metal oxides with varied Li-O bonding beyond TM-O, therefore, both lithium content and transition-metal composition should be taken into consideration in the design of high-capacity cathodes to utilize both conventional transition metal and anionic oxygen redox.

In summary, Li-rich metal oxides, Li_1.2_Ni_0.2_TM_0.6_O_2_ (TM = Mn, Ru), demonstrate high electrochemical capacity, but the redox processes that provide this capacity are distinctly different in each material, even though the structure of each is similar. Using combined X-ray spectroscopy techniques, we illustrated the charge compensation mechanism of transition metals in both compounds and verified the electrochemical activity of Ni and O in LNMO and Ni and Ru in LNRO. In particular, a signature of anionic oxygen redox was directly observed through RIXS mapping of LNMO, whereas this signature was absent in LNRO, indicating that no such oxygen redox was active in LNRO. The unique capability of RIXS on distinguishing the photon emission energies, which is not accessible in sXAS, reveals that the key oxygen feature associated with oxygen redox is intrinsically different from the TM-O hybridization feature in emission energy, although they overlap in the sXAS spectra. By comparing materials with similar structure, our work clearly demonstrated the potential impact of transition metal on the oxygen activation in Li-rich metal oxides. We believe our findings provide additional insights into the complex oxygen redox mechanism in battery electrode beyond the conventional hybridization concept, therefore, expedite the development of advanced high-capacity Li-ion cathodes.

## Methods

### Synthesis of Li_1.2_Ni_0.2_TM_0.6_O_2_ (TM = Mn, Ru)

Li-rich layered oxides were prepared by using Li_2_CO_3_, Ni(OH)_2_, MnCO_3_, and RuO_2_ as precursors, which were purchased from Sigma Aldrich. Precursors at a stoichiometric ratio of Li: Ni: TM = 1.2:0.2:0.6 (TM = Mn, Ru) were first mixed on a Spex 8000 mill for 3 h, followed by a calcination process at a temperature of 950 °C for 15 h in air.

### X-ray diffraction

Powder diffractions were taken at the Advanced Photon Source at Argonne National Laboratory (ANL) on beamline 11-BM (*λ* = 0.459 Å). The beamline uses a sagittal focused X-ray beam with a high precision diffractometer circle and perfect Si(111) crystal analyzer detection for high sensitivity and resolution. XRD patterns were analyzed by the conventional Rietveld method using the general structure analysis system package with the graphical user interface (EXPGUI).^71^

### SEM and TEM characterization

SEM was performed on a JEOL JSM-7000F. Bright field (BF), HRTEM images, and ED patterns were obtained with a JEM-2100F (JEOL) at an accelerating voltage of 200 kV.

### X-ray absorption spectroscopy

Ex situ soft XAS measurements were carried out on beamline 10–1 at the Stanford Synchrotron Radiation Laboratory. Data were acquired under ultrahigh vacuum (10^–9^ Torr) in a single load at room temperature using TEY mode via the drain current and FY mode via a Silicon Photodiodes. RIXS maps were collected in the newly commissioned ultrahigh efficiency iRIXS endstation at Beamline 8.0.1 at the Advanced Light Sources.^72^ All the cycled electrodes were immediately harvested from the cells at designated cutoff voltages to minimize the side reactions between cycled electrodes and electrolyte and vigorously washed by DMC solvents to ensure the removal of soluble surface species. All the dried electrodes were transferred into the experimental vacuum chamber through a specially designed sample transfer kit in an Ar-filled glove box to avoid any air exposure. Sample surface was mounted 45^°^ to the incident beam, and the outgoing photon direction along the RIXS spectrograph is 90^°^. RIXS resolving power and other technical details could be find in our previous report.^73^ Details of RIXS data processing is available in supplementary information. Hard XAS measurements for the Ru K-edge was performed at the Advanced Photon Source on beamline 20-BM-B in transmission mode with electron energy of 7 GeV and average current of 100 mA. The radiation was monochromatized by a Si (111) double-crystal monochromator. Harmomic rejection was accomplished with 20% detuning. For energy calibration, the peak of the first derivative of Ru foil was adjusted to the tabulated value of 22117 eV. XANES and EXAFS data reduction and analysis were processed by Athena software.

### Electrochemical characterization

Electrodes were prepared from slurries containing 80 wt% of active material, 10 wt% of polyvinylidene fluoride (PVdF) binder, and 10 wt% acetylene carbon black (Denka, 50% compressed) in N-methylpyrrolidone solvent. The slurries were casted on carbon-coated aluminum current collectors (Exopack Advanced Coatings) using a doctor blade, and then dried under vacuum at 120 °C overnight. Typical loadings of the active materials were ~2.5 mg cm^–2^. 2032-type coin cells (Hohsen Corp.) containing Li metal, a Celgard 2400 separator, and 1M LiPF_6_ electrolyte solutions in 1:2 w/w ethylene carbonate–diethyl carbonate (Ferro Corporation) were assembled in an Ar-filled glove box (H_2_O <0.1 ppm). Galvanostatic discharge and charge were performed on a Maccor 4200 cycler at C/50 between 4.8 V and 2.0 V. 1C capacity was defined as 250 mA g^–1^.

### Operando DEMS

Operando DEMS measurements were taken on a customized Swagelok type cell connected to a high-pressure gas chromatography valve. The details were described in a previous publication.^74^ The DEMS cell initially rested at open circuit voltage for 6 h and charge/discharge was done under potentiostatic control using a BioLogic SP-300 potentiostat.

### Data availability

The data that support the findings of this study are available from the corresponding authors upon reasonable request.

## Electronic supplementary material

Supplementary Information
