# Supplementary material for: Elucidating anionic oxygen activity in lithium-rich layered oxides
Source: Nat Commun. 2018 Mar 5;9:947. doi: 10.1038/s41467-018-03403-9 (PMC5838240; doi:10.1038/s41467-018-03403-9)
Supplement: Supplementary file 1 — Supplementary Information [file 41467_2018_3403_MOESM1_ESM.pdf]

Supplementary Information

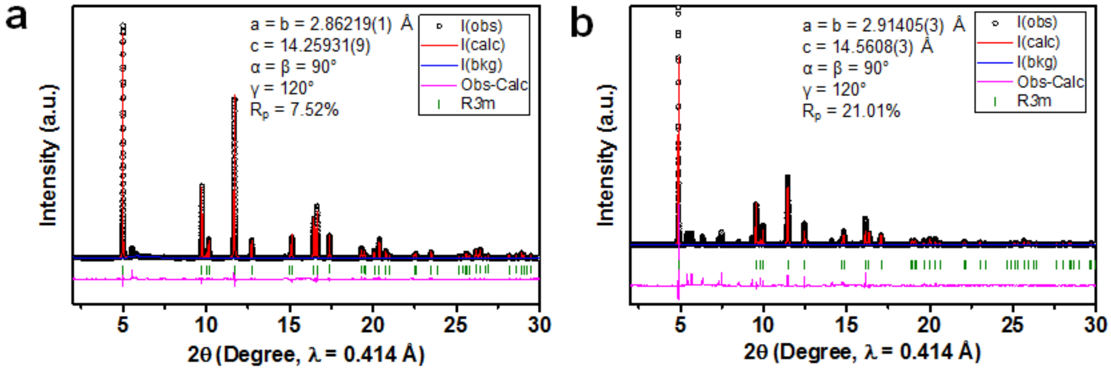

Supplementary Figure 1 Structural characterization of pristine LNMO and LNRO. Synchrotron XRD Rietveld refinement of (a) LNMO and (b) LNRO based on $R\bar{3}m$.

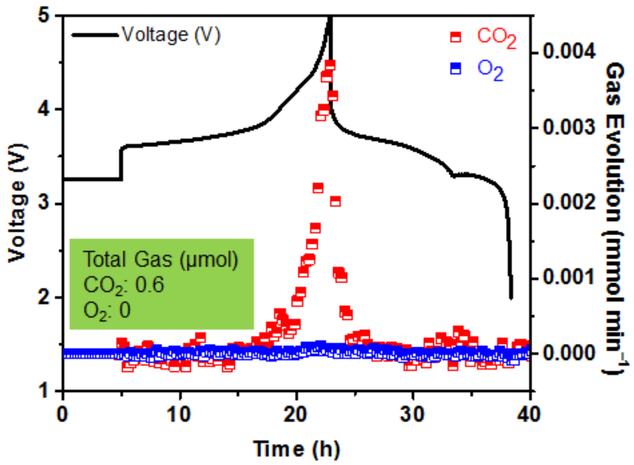

Supplementary Figure 2 Gas evolution of LNRO by operando DEMS. The first cycle voltage profile and gas evolution rates of LNRO. The total active LNRO cathode material used for the measurement was 35.6 mg (316 μmol). Cells were cycled between 5.0 and 2.0 V, at a current density of 10 mA g^{-1}.

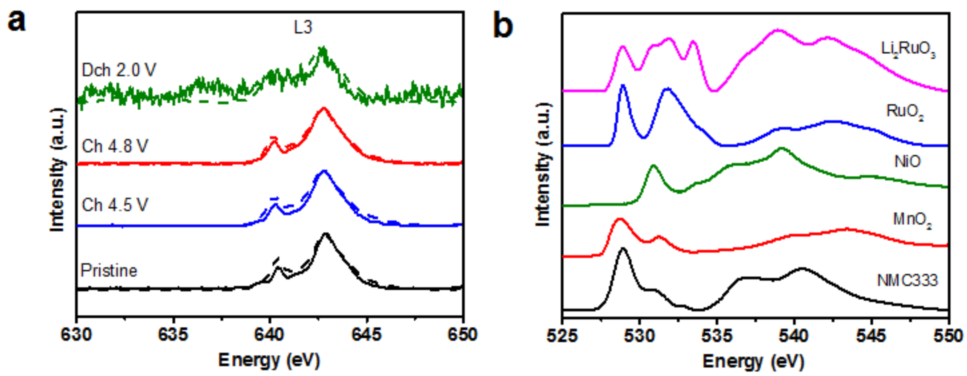

Supplementary Figure 3 Electronic structure of Mn and O as probed by sXAS. (a) sXAS Mn L3-edge spectra of LNMO electrodes in TEY (dash line) and FY (solid line) mode, (b) sXAS O K-edge spectra of reference compounds in TEY mode.

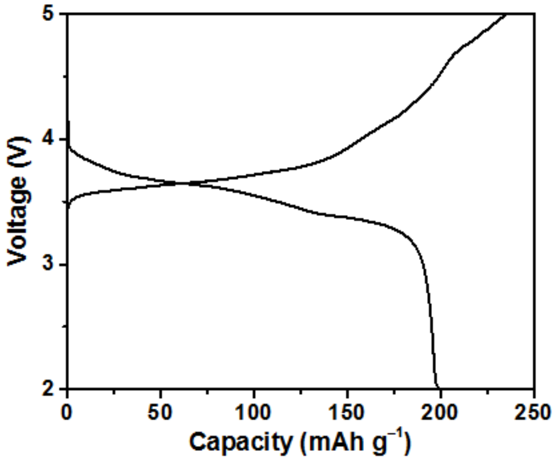

Supplementary Figure 4 First charge-discharge characteristics of LNRO. The first cycle voltage profile of LNRO cycled between 5.0 and 2.0 V, at a current density of 10 mA g^{-1}.

Supplementary Table 1: Refined structural parameters of LNMO based on $C2/m$[a] and $R\bar{3}/m$[b].

LNMO: $C2/m$						
Atom	Wyckoff	x	y	z	Occupancy	Uiso
Mn(1)	4	0	0.1661	0	0.9	0.00773
Ni(1)	4	0	0.1661	0	0.1	0.00773
Li(1)	2	0	0.5	0	0.6	0.01178
Ni(2)	2	0	0.5	0	0.4	0.01178
Li(2)	2	0	0	0.5	1	0.01178
Li(3)	4	0	0.6617	0.5	1	0.01178
O(1)	4	0.219	0	0.226	1	0.0076
O(2)	8	0.2533	0.3238	0.2231	1	0.0076

[a]LNMO lattice parameter: a = 4.95747(2) Å, b = 8.58634(2) Å, c = 5.03250(2) Å, α = γ = 90°, β = 109.1695°, R_p = 8.17%.

LNMO: $R\bar{3}/m$						
Atom	Wyckoff	x	y	z	Occupancy	Uiso
Li(1)	3	0	0	0	0.9756	0.01013
Ni(2)	3	3	0	0	0.0245	0.01013
Li(2)	3	0	0	0.5	0.2244	0.00136
Ni(1)	3	0	0	0.5	0.1756	0.00136
Mn(1)	3	0	0	0.5	0.6	0.00136
O(1)	6	0	0	0.241472	1	0.00888

[b]LNMO lattice parameter: a = b = 2.86219(1) Å, c = 14.25931(9) Å, α = β = 90°, γ = 120°, R_p = 7.52%.

Supplementary Table 2: Refined structural parameters of LNRO based on *C*2/*c*[a] and $R\bar{3}/m$[b].

LNRO: *C*2/*c*						
Atom	Wyckoff	x	y	z	Occupancy	Uiso
Ru(1)	8	0.2471	0.0828	0.0029	0.9	0.01136
Ni(1)	8	0.2471	0.0828	0.0029	0.1	0.01136
Li(1)	4	0.25	0.25	0.5	0.6	0.0057
Ni(2)	4	0.25	0.25	0.5	0.4	0.0057
Li(2)	4	0	0.0837	0.25	1	0.00393
Li(3)	4	0	0.4154	0.25	1	0.00355
Li(4)	4	0	0.75	0.25	1	0.00545
O(1)	8	0.1231	0.2496	0.1174	1	0.00563
O(2)	8	0.1231	0.5852	0.1174	1	0.00052
O(3)	8	0.1231	0.9173	0.1174	1	0.01282
RuO_2: *P*42/*mnm*						
Ru(1)	2	0	0	0	1	0.00013
O(1)	4	0.30346	0.30346	0	1	0.00013

[a]LNRO lattice parameter: a = 5.0369(6) Å, b = 8.75352(8) Å, c = 9.85535(6) Å, α = γ = 90°, β = 99.844°; RuO_2 lattice parameter: a = b = 4.49204(5) Å, c = 3.10687(7) Å, α = β = γ = 90°; R_p = 9.71%.

LNRO: $R\bar{3}/m$						
Atom	Wyckoff	x	y	z	Occupancy	Uiso
Li(1)	3	0	0	0	1	0.00023
Li(2)	3	0	0	0.5	0.2	0.00838
Ni(1)	3	0	0	0.5	0.2	0.008
Ru(1)	3	0	0	0.5	0.6	0.008
O(1)	6	0	0	0.243217	1	0.00513

[b]LNRO lattice parameter: a = b = 2.91405(3) Å, c = 14.5608(3) Å, α = β = 90°, γ = 120°, R_p = 21.01%.

Supplementary Note 1. Supplementary **Figure 1** shows the Rietveld refinement of LNMO and LNRO based on $R\bar{3}m$ symmetry. As shown in Supplementary **Figure 1a**, a good fit was obtained for LNMO refinement as the weak superstructure peaks in the 2θ region of 5 to 8 do not significantly affect the refinement results. But, LNRO refinement based on $R\bar{3}m$ symmetry did not result in a good fit (Supplementary **Figure 1b**), which was largely due to the mismatch in the region of superstructure peaks because of their relatively high intensities.

Supplementary Note 2. Supplementary **Figure 2** shows the first cycle voltage profile and gas evolution rates of LNRO between 5.0 and 2.0 V. No oxygen evolution was detected, instead, more CO_2 evolution was observed, 0.6 μmol at 5 V (*vs.* 0.1 μmol at 4.8 V). But this was significantly lower compared to the total gas evolution from LNMO at 4.8 V, as evidenced by 6.5 μmol CO_2 and 10.7 μmol O_2.

Supplementary Note 3. Supplementary **Figure 3a** shows sXAS Mn L3-edge spectra of LNMO at various states of charge. No major change in Mn L3-edge was detected during the charge, except a small reduction at the end of discharge. **Supplementary Figure 3b** shows sXAS O K-edge of reference compounds, indicating the hybridization peaks affected by both Ni/Mn*3d* and Ru*4d* states are overlapping with the O*2p* states.

Supplementary Note 4. LNRO was charged to 5 V to explore additional oxygen redox activity at a higher potential. As shown in Supplementary **Figure 4**, further charging LNRO to 5.0 V did not result in an increase in the capacity, suggesting no additional redox activity even at 5 V. Instead, a slight decrease in discharge capacity likely due to a detrimental effect of high voltage on LNRO sample.

Supplementary Methods

RIXS data processing. The data used to generate the RIXS maps were collected in the following steps: (1) sXAS were first collected on each sample to define the excitation energy range and calibrate the energy values; (2) at each excitation energy with a step size of 0.2 eV across the whole O K near edge regime, the diffracted fluorescence photons were collected in the form of 2D image by the Andor CCD detector on the spectrograph; (3) cosmic ray signals were determined and deducted from the raw 2D images; (4) intensity of the raw 2D image was directly integrated along the energy channels to generate a single RIXS spectra, corresponding to the particular excitation (incident beam) energy; (5) the integrated 1D spectrum was normalized to both the incident beam flux monitored real-time during the data collection and collection time (in seconds); (6) repeat step (2) – (5) to collect and process the RIXS spectra at all the relevant excitation energies, then combine all the processed spectra into a 2D image map, as shown in the figures; (7) the energy values of the RIXS maps are finally calibrated by following the elastic features (with the same energy on excitation and emission photons) at multiple energies. Of note, the colour scale has been tuned in the figures to emphasize the contrast of intensity. Because the central discussions in this manuscript do not require quantitative analysis of the RIXS line shape, no energy distribution curves (EDCs) were plotted in this paper.
